# DsRNA as pathogen-associated molecular pattern in innate immunity and multiple functions of the RNAi machinery complicate the use of RNAi in pest control

**DOI:** 10.3389/finsc.2025.1749008

**Published:** 2026-01-16

**Authors:** Min Feng, Jisheng Liu, Luoluo Wang, Luc Swevers

**Affiliations:** 1 Guangdong Provincial Key Laboratory of Agro-animal Genomics and Molecular Breeding, Guangdong Sericulture Engineering Research Center, College of Animal Science, South China Agricultural University, Guangzhou, China; 2 School of Life Sciences, Guangzhou University, Guangzhou, China; 3 National Key Laboratory of Green Pesticide, College of Plant Protection, South China Agricultural University, Guangzhou, China; 4 Insect Molecular Genetics and Biotechnology, Institute of Biosciences and Applications, National Centre for Scientific Research “Demokritos”, Athens, Greece

**Keywords:** dsRNA, exo-RNAi, innate immunity, PAMP, siRNA pathway

## Abstract

In contrast to mammals, insects possess a separate machinery for processing of long dsRNAs into siRNAs for the cleavage of viral RNAs. The process of RNAi is considered very efficient in all insects once the delivery in the cytoplasm occurs such as during RNA virus replication. For the application of RNAi as insecticide to succeed, efficient uptake of intact dsRNA into the cytoplasm therefore is necessary, which seems to occur by natural mechanisms in the leaf beetles for which RNAi-based insecticides already have been marketed. In most insects, relatively high amounts of dsRNA are required to trigger gene silencing which raises questions regarding potential side effects. Besides, RNAi is considered as the major antiviral defense mechanism, at least in *Drosophila*, but not necessarily in all other insects. Following increasing evidence from the recent literature, it has become prudent to include the sensing of dsRNA as an immune trigger to evaluate the extent of the RNAi mechanism that is triggered by dsRNA. In this review, an overview of mechanisms is presented regarding how the recognition of dsRNA as a “pathogen-associated molecular pattern”, the multiple additional functions of the canonical siRNA factors and the modulation of the function of Dicer-2 and Ago-2 by dsRNA-binding proteins may complicate the efficiency of the exo-RNAi process and aggravate its application for pest control.

## 1 Introduction

### 1.1 Historical background

Since its discovery, the process of RNA interference (RNAi), as a method of gene silencing, has attracted keen interest for applications in medicine (1). Since nucleic acids are polar molecules of relatively high molecular weight, which prevents the crossing of the cellular plasma-membrane, it nevertheless took almost twenty years of engineering, mainly focused on the improvement of delivery vehicles, before the first RNAi-based drugs became available (2). Applications in livestock and crops were more straightforward because of the availability of transgenic technologies that allowed the intracellular production of molecules with double-stranded RNA (dsRNA) structure that were designed to provide protection against pathogens or re-program metabolic and developmental pathways (3, 4). Among the applications of RNAi, the use of biocidal dsRNA is the most remarkable and was only possible by the discovery that particular organisms, belonging to the taxa of fungi, nematodes and beetles, can be very sensitive to the uptake of dsRNA in the environment for the molecular regulation of physiological functions (5–7).

### 1.2 Core mechanisms and pathways: the siRNA pathway as an antiviral defense mechanism

In contrast to mammals, but similarly to plants, insects possess a separate machinery for processing of long dsRNAs into small RNAs (8, 9). This distinct pathway, called the short interfering RNA (siRNA)-pathway, is considered the major antiviral defense pathway in insects because of its efficient processing of long dsRNA molecules that are typically encountered as replication intermediates during replication of RNA viruses (10–12). The Dicer-2 (Dcr-2) enzyme of the siRNA pathway shows signs of strong positive selection, conform to its involvement in the virus-host arms’ race (13), and effectively processes long dsRNA, in contrast to precursor microRNA (pre-miRNA), which is the preferential substrate for Dcr-1 (14). MiRNAs and siRNAs also form distinct RNAi-induced silencing complexes (RISCs) associated with the Argonaute proteins Ago-1 and Ago-2, respectively, consistent with their functional divergence (15).

### 1.3 Early uses and concepts of RNAi in pest control

Because of the feature of intracellular defense against viral infection, the process of RNAi is considered to be very efficient in all insects once the delivery in the cytoplasm occurs (16; note, however, that important differences exist when dsRNA is applied extracellularly; see section 4). It is generally considered that the activation of the (antiviral) siRNA pathway represents the major mechanism by which insecticidal dsRNAs induce gene silencing and toxicity in applications of RNAi-mediated pest control (17–21). Indeed, dsRNA is administered with the purpose of the silencing of essential genes in the cells and organism, thereby triggering physiological and developmental disruption and resulting in increased mortality. In such scheme, the antiviral siRNA pathway is hijacked and re-directed to cellular mRNAs, reminiscent of the generation of auto-immunity. The process of viral infection, however, mainly results in the intracellular production of dsRNA, with more immediate access to the RNAi machinery. By contrast, the application of insecticidal dsRNA by necessity occurs in the environment (environmental RNAi; 22). The major differences in sensitivity to environmental or systemic RNAi among insects therefore are caused by extracellular degradation of dsRNA, e.g. in the gut, in combination with defective uptake mechanisms (23). Advances in RNAi-based pest control have therefore focused on the improved delivery of dsRNA molecules across structural and physiological barriers into the cytoplasm of the cells in target pest insects (e.g. by nanoparticles; 20, 23).

### 1.4 Integration of the RNAi mechanism in a broader view of the regulation of innate immunity

In model organisms such as *Drosophila* and mosquitoes, recent research has revealed the importance of dsRNA as a trigger of innate immunity pathways, beyond the RNAi mechanism. In addition, Dicer-2 and Ago-2 can have pleiotropic functions in the regulation of stress and immunity that extend beyond the canonical siRNA pathway. It is therefore timely to organize these newly developed concepts and provide a solid background for further research. This review should stimulate more detailed investigations of the RNAi mechanism in insect pest populations that take into account factors such as the activation of other innate immune pathways by dsRNA and the presence of covert pathogen infections and stress conditions that could affect RNAi.

The review first investigates the prevalence of RNAi as an antiviral defense mechanism in *Drosophila* (section 2) followed by an assessment of its lower relative importance in Lepidoptera (section 3). It can be concluded that the siRNA pathway may not be the most important antiviral pathway in Lepidoptera for which also evidence exists in mosquitoes (conclusion of section 3). The deficiency of the siRNA pathway in Lepidoptera against viruses may reflect on its relative inefficiency in systemic and environmental RNAi experiments (24). By contrast, there is overwhelming evidence that insecticidal effects of environmental dsRNA are caused by an RNAi mechanism in leaf beetles (section 4) while the importance of the siRNA pathway during antiviral defense in these beetles has not been examined. The latter is an important knowledge gap which needs to be addressed in future research (see discussion in section 8). The review then examines in more detail the deviations/imperfections that can occur in the canonical siRNA pathway as it is assumed (section 5), the non-RNAi function of Dcr-2 in the regulation of translation (section 6), and the recent accumulating evidence that dsRNA can stimulate other innate immune pathways than RNAi (section 7). Interestingly, Dicer-2 increasingly emerges as a hub for the regulation of stress and immune pathways (section 6) and the activation of dicer activity is dependent on dsRNA structural features (section 7.2 and 7.3). In the discussion (section 8), an attempt is made how the new knowledge can be applied for the improvement of the application of insecticidal dsRNA in field applications, primarily with respect to off-target effects in the targeted pest as well as hitherto unexpected effects on non-target (beneficial) organisms.

## 2 Dogma: the siRNA pathway as the major antiviral defense mechanism in *Drosophila*

The importance of the siRNA pathway as an antiviral defense mechanism was first demonstrated in *Drosophila* (11, 12). Crucial was the observation of enhanced viral susceptibility and replication in flies deficient for Dcr-2 or Ago-2 as well as the discovery of viral suppressors of RNAi (VSRs) in the viral genomes (10, 25–27). Because Dcr-2 apparently has evolved to process long dsRNA molecules, its antiviral function may have been primarily directed against infections with RNA viruses (28).

The siRNA pathway was also proposed as an antiviral mechanism against DNA viruses, despite the expected much lower production of dsRNA trigger molecules (29). Nevertheless, an antiviral role against DNA viruses was demonstrated, triggered by dsRNA molecules that are formed by hybridization of complementary transcripts or intramolecular hairpin structures (29–31). VSR genes were identified in genomes of DNA viruses such as Heliothis virescens ascovirus HvAV-3e (32) and Invertebrate iridescent virus 6 (IIV-6; *Iridoviridae*) (33, 34).

Interestingly, the preferential targets of viral siRNAs (vsiRNAs) are poly-adenylated viral mRNAs in comparison to viral genomic or antigenomic RNAs (35). During infection with GFP reporter viruses, administration of dsRNA targeting GFP effectively silences the expression of GFP transcripts while it does not affect virus replication, which was observed both for RNA viruses with positive-strand and negative strand genomes (36). Ago-2 protein is also found to be associated with ribosomes, presumably to monitor incoming transcripts for degradation, indicating a significant presence of RISC complexes at the sites of translation (36).

By contrast, an important role of miRNAs in the control of virus infections in *Drosophila* was considered much less likely (11, 37, 38). With respect to the third major small RNA pathway that involves the production of Piwi-associated RNAs (piRNAs), which are implicated in anti-transposon defense and are generated by a Dicer-independent mechanism (39), it was observed that no viral piRNAs were produced during infection, leading to the conclusion that the piRNA mechanism is not involved in antiviral defense in *Drosophila* (40).

*Drosophila* has functioned as the major model system in the research of RNAi as an antiviral mechanism. However, it is increasingly realized that interactions of insect hosts with their viruses have unique characteristics and that the contribution of RNAi to antiviral defense, together with the participation of other innate immune pathways, needs to be evaluated separately for each insect-virus combination (38). In addition, because of the co-evolution between hosts and pathogens (the latter including transposons that are also controlled by RNAi, albeit with mechanisms that differ in details) as a dynamic struggle, it can be expected that the RNAi machinery has undergone lineage-specific modifications with consequences for its regulation and function. Success in the exploitation of the siRNA pathway for pest control by insecticidal dsRNAs therefore needs to take into account the evolution of the RNAi pathway as a mechanism of defense against parasitic nucleic acids.

## 3 How important is RNAi as an antiviral mechanism in Lepidoptera?

As an example of the importance of the siRNA pathway in antiviral defense in non-drosophilid insects, its contribution to innate immunity against virus infection is discussed for Lepidoptera. In lepidopteran insects, a variety of both DNA and RNA virus infections can be detected (41, 42) but, because of its economic importance, most research on viruses has been carried out in the domesticated silkworm, *Bombyx mori* (43, 44), with a focus on the RNA virus Cypovirus (BmCPV, *Reoviridae*; 45) and the DNA virus Bombyx mori nucleopolyhedrovirus (BmNPV; *Baculoviridae*) (46, 47). Relevant information from research with other baculoviruses with respect to the RNAi response will also be included.

### 3.1 BmCPV

During BmCPV infection of the larval midgut, the core siRNA genes encoding Ago-2 and Dcr-2 become upregulated (48) although this was not observed in another study (49). In addition, vsiRNAs of 20 nt size are produced of which the abundance correlates with the infection severity (48, 50). Transgenic silkworm strains that express RNA hairpins (with dsRNA structure) targeting multiple BmCPV genes show increased resistance against BmCPV infections (51). However, no knockdown or knockout experiments of Dcr-2 and Ago-2 were reported while the presence of VSRs also has not been explored extensively (45). In BmN cells, it was proposed that the non-structural protein NSP8 can function as a VSR during BmCPV infection through its interaction with Ago-2 (52). However, few vsiRNAs are produced during infection of BmN cells, indicating a weak RNAi response, and the function of NS8 as VSR remains to be confirmed during midgut infection in larvae. In the absence of much additional evidence, it remains to be validated whether a canonical RNAi mechanism is functional in the insect tissues: for instance, Dicer-2, as an endonuclease that cleaves essential viral replication intermediates, potentially could provide antiviral defense in the absence of loading siRNAs into Ago-2 and RISC formation (10).

Most experiments with BmCPV have focused on the transcriptional response induced by infection (48, 53) as well as the role of both viral and host miRNAs that regulate infection dynamics (54–57) and the interaction with long non-coding RNAs (lncRNAs; 58), including circular RNAs (circRNAs; 59, 60). Interestingly, BmCPV circular RNAs encode peptides that negatively regulate viral replication and activate the immune response (61, 62).

Research on the involvement of miRNAs and circRNAs provided additional information on the involvement of the siRNA factors Ago-2 and Dcr-2 on BmCPV replication. The prevalence of the miRNA pathway over the siRNA pathway in antiviral defense was suggested in one report by the observation that BmCPV replication was repressed in silkworms silenced with Dcr-1 (miRNA pathway) while it was stimulated by knockdown of Dcr-2 (63). Furthermore, BmCPV upregulates the host lncRNA *Linc20486* which promotes infection through the inhibition of the siRNA genes *Dcr-2* and *Ago-2* and the miRNA gene *Dcr-1* (but not *Ago-1*) while the expression of autophagy-related genes and selected innate immunity genes was not affected (64). The siRNA pathway is also activated by the production of a viral circular DNA, vcDNA-S7, which is formed by a reverse transcriptase mechanism using the dsRNA segment S7 of the BmCPV genome as template (65). Transfection of vcDNA-S7 in BmN cells induces the expression of *Dcr-2* and *Ago-2* and results also in the production of vsiRNAs, albeit at low level (65). The observed process is reminiscent of a mechanism of amplification of siRNA production in *Drosophila*, where viral DNA forms function as templates to produce secondary vsiRNAs against RNA viruses (66, 67).

Although viral small RNAs with the size of piRNAs (26–30 nt) were identified during BmCPV infection of silkworm midgut, these were not considered as “true” vpiRNAs because of the absence of 1U bias and ping-pong signature (50). While “true” vpiRNAs were reported in persistent virus infections in lepidopteran cell lines in some cases (68) and manipulation of expression of Siwi and BmAgo-3 can affect infection levels of RNA viruses (69), the role of the piRNA pathway in antiviral defense remains contentious in Lepidoptera and may involve PIWI proteins but not always piRNAs (69) (see section 5 for additional discussion).

In Sf21 cells, derived from *Spodoptera frugiperda*, expression of the BmCPV capsid shell protein (CSP) resulted in the induction of expression of *Dcr-2* (but not *Ago-2*) (70). In addition, BmCPV virions isolated from polyhedra and virus-like particles (VLPs) based on BmCPV capsid proteins were able to induce the transcriptional response of the siRNA genes *Dcr-2* and *Ago-2* (together with miRNA genes and other immune genes) in midgut tissue of silkworms *ex vivo* (71). Such experiments indicate the detection of BmCPV capsid proteins as “pathogen-associated molecular patterns” (PAMPs) by Sf21 cells and midgut tissue to activate the expression of RNAi and other immune genes.

### 3.2 Baculovirus – BmNPV

During infection of *Helicoverpa zea* fat body (HzFB) cells with *H. armigera* single NPV (HaSNPV, group II alphabaculovirus), abundant vsiRNAs were detected that were mapped to hot spots in the viral genome (72). Most vsiRNAs mapped mostly to the strand corresponding to the sense strand of the viral genes, indicating that expression levels and RNA secondary structures are major substrates for vsiRNA production (72). For Autographa californica multiple NPV (AcMNPV), a group I alphabaculovirus, however, vsiRNAs mapped equally to both strands while also hot spot and cold spot regions were detected (73). Importantly, p35 protein of AcMNPV was identified as a powerful VSR and suppression of RNAi could be separated from the function of inhibition of apoptosis (73).

Silencing of *Dcr-2* (and not *Dcr-1*) increased viral replication and the RNA levels of viral genes corresponding to the hot spots during HaSNPV infection (72). During AcMNPV infection of Sf9 cells, both *Dcr-2* and *Ago-2* were modestly induced and silencing of *Dcr-2* and *Ago-2* increased the replication of AcMNPV lacking *p35* (74).

During oral infection of BmNPV, induction of *Dcr-2* was observed in the midgut and hemocytes. Knock-down of *Dcr-2* also resulted in a modest increase in the levels of viral genomic DNA (75). However, in other studies, variable effects on the expression of *Dcr-2* and *Ago-2* were observed following oral infection of both BmNPV and Bombyx mori Bidensovirus (BmBDV; *Bidnaviridae*; a single-stranded DNA virus) that were also found to be dependent on the silkworm strain (49, 76).

Baculovirus infection results in both the expression of virus-encoded miRNAs and a significant change in the expression of cellular (host) miRNAs (77–79). Many research efforts are currently devoted to the mechanisms by which baculovirus-encoded miRNAs and differentially expressed cellular miRNAs regulate the infection cycle and the immune response, by identifying mRNA targets (80, 81; 82) and by investigating interactions with lncRNAs and circRNAs (83–85). Over-expression of artificial miRNA precursors against essential baculoviral genes has been used as an antiviral mechanism against BmNPV in silkworm (86).

Small RNA sequencing indicated that, during BmNPV infection of silkworm fat body and midgut tissues, no vpiRNAs were produced, based on criteria such as 1U bias and ping-pong signal (87). On the other hand, a large amount of host-derived piRNAs became differentially expressed and putative targets in midgut and fat body were predicted by bio-informatics analysis (87). Given the increasing role of piRNAs in the regulation of cellular functions such as stem cell maintenance, genome re-organization, inflammation, ageing and cancer (88–91), an interesting question relates to whether piRNAs could contribute to the reprogramming of the host cells during infection. Surprisingly, silencing and over-expression experiments indicated that Siwi and BmAgo-3 could promote BmNPV infection (92), in contrast to their (contentious) antiviral role against RNA viruses (69).

### 3.3 Conclusion

While there are clear indications for a role of the siRNA pathway, mediated by Dcr-2 and Ago-2, in antiviral defense against both RNA and DNA viruses in Lepidoptera, it is equally apparent that other RNAi mechanisms are involved, particularly miRNAs. In addition, non-RNAi innate immunity functions in antiviral defense which is particularly well documented for baculovirus (nucleopolyhedrovirus) infections. Other antiviral pathways include the “classical” IMD and Toll pathways, originally identified for antibacterial and antifungal defense, the Jak-STAT pathway for repair of tissue damage, the extracellular prophenoloxidase/melanization cascade, the induction of apoptosis and autophagy and the recently identified cGAS/STING antiviral defense mechanism (46, 93, 94) (see also section 7). In model insects, such as *Drosophila* and *Aedes* mosquitoes, a difference was observed with respect to the relative importance of the siRNA pathway during systemic versus oral infections (initiated by hemolymph injection versus feeding). More specifically, the siRNA pathway may not be able to protect the midgut against virus infection in mosquitoes (95) while viral infections can also be cleared in the mosquito midgut in the absence of the antiviral activity of the siRNA pathway (96). Antiviral pathways in the midgut of mosquitoes (and *Drosophila* as well) include Toll and Extracellular signal-Regulated Kinase (ERK) signaling and the interaction with the microbiota (97–100).

## 4 Insecticidal activity of dsRNA for control of the western corn rootworm occurs through the exo-RNAi pathway

In insects and other eukaryotes, the siRNA pathway is not only activated by dsRNA originating from invading virus infections or taken up from the extracellular medium (for cells/organisms that have this capacity) but also from loci in the cellular genome that produce structured RNAs (e.g. hairpins) or overlapping complementary transcripts from transposons, repetitive elements and cellular genes (101). These processes are called exogenous RNAi (exo-RNAi) and endogenous RNAi (endo-RNAi), respectively, according to the source of dsRNA.

### 4.1 High sensitivity of chrysomelid beetles to environmental RNAi facilitated the development of dsRNA as insecticide

Among insects, chrysomelid beetles such as the western corn rootworm, *Diabrotica virgifera*, and the Colorado potato beetle, *Leptinotarsa decemlineata*, are very sensitive to environmental RNAi (102, 103), which means that these insects can efficiently internalize dsRNA from the environment (food) and activate the exo-RNAi pathway. Abundant evidence exists that the uptake of insecticidal dsRNA is processed by the canonical exo-siRNA pathway, characterized by dicing by Dcr-2 and slicing by Ago-2, in the chrysomelid beetles, which will be discussed in some detail for *Diabrotica*. The sensitivity is illustrated by the low median lethal concentration (LC_50_) values of 1–5 ng dsRNA/mL of diet, recorded after a period of 12 days (5). Evidence for a canonical siRNA process is the observation of uptake of fluorescent dsRNA by the midgut, the processing of dsRNA to 21 nt siRNAs, the decline of target mRNA by > 20-fold and the dependence on Dcr-2 and Ago-2 (102, 104, 105). Importantly, the observed phenotype agreed with the hypothesized function of the targeted genes: in the case of feeding of dsDvSnf7, belonging to the ESCRT-III complex involved in vesicle trafficking, impairment of autophagy, accumulation of ubiquitinated proteins, disruption of acidic lysosomal activity and de-stabilization of membranes is observed, all contributing to the induction of lethality (106, 107). Feeding of dsRNA to the beetle larvae and adults also causes systemic effects, i.e. spreading to internal tissues of plant-derived dsRNAs and small RNAs (102) which can even extend to the next generation (i.e. parental RNAi; 108). The effects of dsRNA are dose-dependent and proportional to the number of siRNAs that are produced (109) and no amplification mechanism resulting in the generation of “secondary” siRNAs (as observed in plants and the roundworm *C. elegans*) could be detected (110). The high sensitivity to environmental RNAi is considered to be a crucial factor for the successful development of dsRNA insecticides against the western corn rootworm *D. virgifera* (as a plant protection trait; 111) and the Colorado potato beetle *L. decemlineata* (as a sprayable product; 112).

### 4.2 Comparative sensitivity to environmental RNAi among major insect orders

The differences in sensitivity to ingested dsRNA between Coleoptera and other insects (Lepidoptera, Diptera, Hymenoptera, Hemiptera, Isoptera, Orthoptera) were for the first time summarized in a landmark publication (113) and its general conclusion has withstood the test of time (e.g. 114, 115). Thus, while the sensitivity of coleopterans to environmental dsRNA is measured as “parts per billion” (ppb), much higher concentrations are needed for the other insect orders (“parts per million” or ppm), i.e. differences of 100-1,000-fold (113). During the last 15 years many experiments were conducted to determine the sensitivity to environmental RNAi among insect pests as well as the mechanisms that are responsible for the differences in sensitivity. In such experiments, dsRNAs were typically administered after injection into the hemolymph (in this review referred to as “systemic RNAi”) as well as after feeding (“environmental RNAi”) (22). In both cases, the stability of dsRNA in the hemolymph/environment and the efficient uptake of dsRNA from the extracellular medium into the cytoplasm are considered crucial for the success of the use of RNAi in pest control (19, 116). An overview, in general lines, of the differences in sensitivity with respect to systemic and environmental RNAi among insects of different orders is presented in **Table 1**. Such classification of variability is by necessity approximate since among insects within the same order already large differences can be apparent (e.g. 129). Regarding hemipterans, leafhoppers were found to be more sensitive to RNAi than aphids (116). Within Coleoptera, not all species are very sensitive and many beetles require much higher concentrations of dsRNA to achieve gene silencing than in the case of *Diabrotica* and *Leptinotarsa* (130).

**Table 1:** Comparative sensitivity to systemic and environmental RNAi among major insect orders.

Insect order	General sensitivity to extracellular dsRNA	Main mechanisms (selection)	References (selection)
Coleoptera	Systemic: high Environmental: high	Low dsRNA degradation in gut Efficient uptake of dsRNA in the cytoplasm	Spit et al. (117) Koo and Palli (118)
Diptera	Systemic: tissue specific Environmental: variable	Relatively high susceptibility of hemocytes Variable uptake of extracellular dsRNA	Miller et al. (119) Taning et al. (120)
Hemiptera	Systemic: moderate Environmental: low to moderate	DsRNA degradation in saliva Limited uptake of dsRNA in the cytoplasm	Christiaens et al. (121) Jain et al. (122)
Hymenoptera	Systemic: variable Environmental: low to moderate	Tissue specific effects of efficiency Modulation by virus infections	Yang et al. (123) Zhang et al. (124)
Lepidoptera	Systemic: low Environmental: low	High dsRNase activity in the gut Limited uptake of dsRNA in the cytoplasm	Shukla et al. (125) Yoon et al. (126)
Orthoptera	Systemic: high Environmental: low	High dsRNase activity in the gut Efficient uptake of dsRNA in the cytoplasm	Wynant et al. (127) Wynant et al. (128)

Systemic and environmental RNAi refer to administration of dsRNA by injection or feeding, respectively. It is noted that the mentioned trends are to a large extent tentative and that relatively large differences can also occur within an insect order. Experimental results can show considerable variability among laboratories which could be explained by variations in strains of experimental insects, developmental stages, rearing conditions and methods of application. Taking into account these caveats, the Table nevertheless is believed to reflect the general expectation for success with RNAi within each major order. Regarding mechanisms, most are based on a good dose of speculation since in-depth investigations were not performed in many instances. The list of references represents only a very small part of the huge research efforts in this field but will provide useful information for the interested reader.

### 4.3 RNAi versus non-RNAi effects of dsRNA?

The low doses of dsRNA to trigger environmental RNAi in *Diabrotica* and *Leptinotarsa* leave little doubt that a canonical siRNA pathway causes the insecticidal effects in chrysomelid beetles. In other insects, there is less certainty for the (sub)lethal effects of dsRNA being caused by only RNAi (i.e. specific gene silencing) since in many experiments mortality or disruptions in development and reproduction are only recorded at relatively high doses.

**Figure 1:**
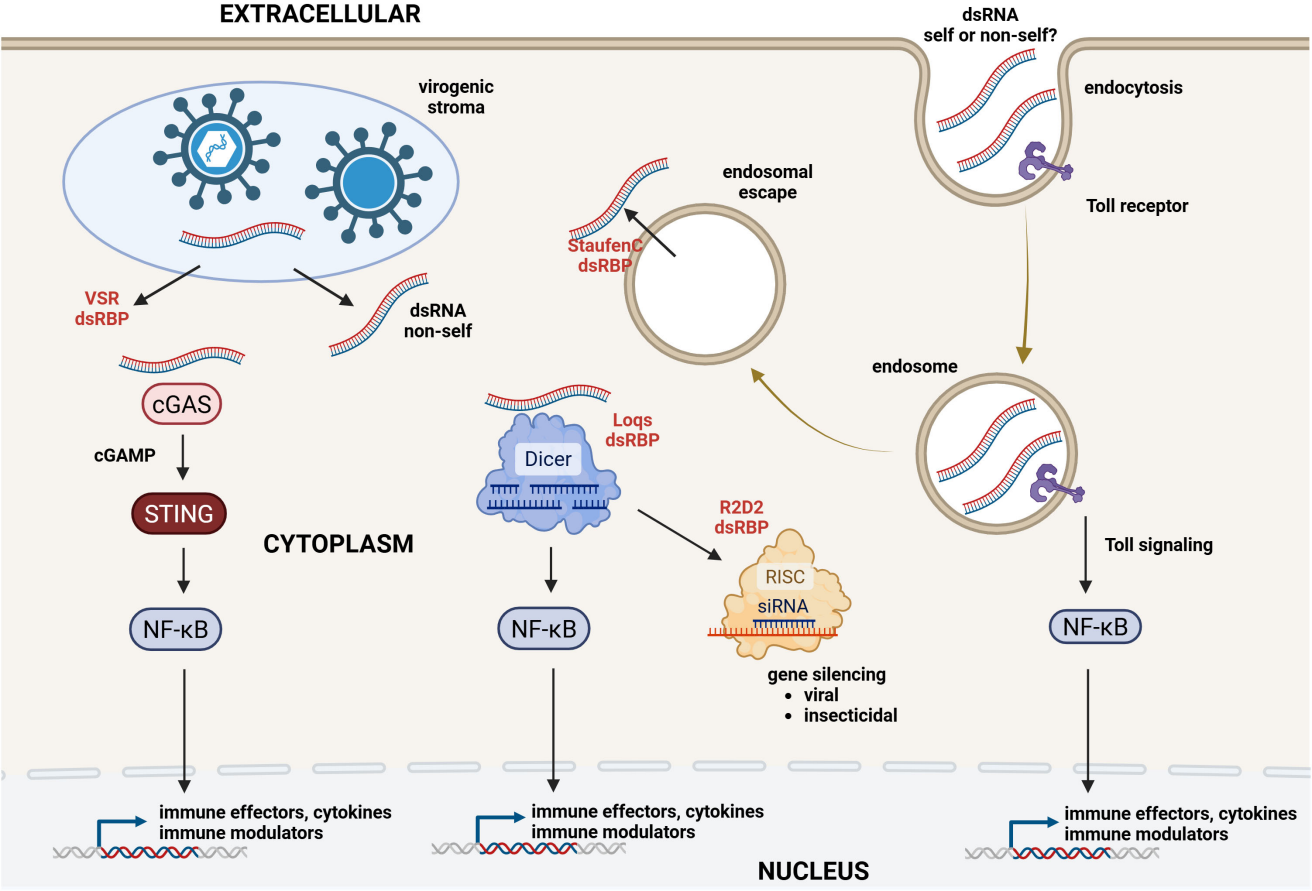
Signaling/processing pathways of dsRNA in insect cells. Insecticidal dsRNA is taken up by endocytosis while dsRNA production by RNA viruses occurs in the cytoplasm. Within endosomes, dsRNA as PAMP hypothetically could bind PRRs such as Toll to activate NF-κB transcription factors that induce expression of immune-related genes. Both insecticidal dsRNA and viral dsRNA can be released in the cytoplasm by endosomal escape and accidental release from the virogenic stroma, respectively. In the cytoplasm, dsRNA as PAMP hypothetically can interact with PRRs such as cGAS and the helicase domain of Dcr-2 to activate NF-κB transcription factors that induce expression of immune-related genes. In another pathway, dsRNA is processed by Dcr-2 into siRNAs that become incorporated in the RISC complex for the induction of specific gene silencing (RNAi mechanism for toxic effects of insecticidal dsRNA and for antiviral defense). The figure also highlights the importance of dsRNA-binding proteins (dsRBPs): StaufenC mediates the endosomal escape of dsRNA, Loqs aids in the interaction of dsRNA containing imperfect ends with Dicer, R2D2 is involved in the loading of RISC with siRNAs and VSRs (viral suppressors of RNAi) can sequester dsRNA and siRNA. DsRNA can have features that are recognized as “self” or “non-self” which could affect its interaction with sensors and processing. Another source of dsRNA (with “self” features) consists of repetitive elements, transposons and structured loci that reside in the genome but such “endogenous” dsRNAs do not activate an immune response in normal physiological conditions (not shown in the figure). Created in https://BioRender.com.

In most RNAi experiments involving insects, production of siRNAs together with the operation of an intact siRNA pathway is only rarely investigated (131). Survival curves and graphs representing changes in life cycle traits and fecundity and hatching rates are commonly presented in the absence of details whether the knockdown corresponds to the phenotype at the cellular and molecular level. Knockdown of the targeted gene is typically always recorded in comparison with a non-specific dsRNA but it should be realized that this can occur by the activation of both specific processes (i.e. sequence-specific RNAi) and non-specific potential side-effects. Even for sequences that target specific regions, large differences in efficiency of silencing are prevalent, for reasons that are not completely understood (132). Recently, it was found that dsRNA can act as a virus infection-associated PAMP to activate other pathways in innate immunity in addition to RNAi (133, 134) (see section 7 and **Figure 1**). In addition, Dcr-2 and Ago-2 have additional functions than RNAi and regulate heterochromatin stability, immune and stress gene expression and cytoplasmic poly-adenylation (135, 136) (see sections 5–7 and **Table 2**). The observations that dsRNA is not merely an RNAi substrate and can activate signaling pathways as a PAMP and that *Dcr-2* and *Ago-2* are pleiotropic genes invite detailed analysis with respect to the effects of administration of dsRNA to insect pests and to what extent a *bona fide* RNAi mechanism is triggered. Actually, any aberration in the siRNA pathway could have significant effects on RNAi efficiency.

**Table 2:** Overview of the non-RNAi functions of the siRNA factors Dcr-2 and Ago-2.

Species	Non-RNAi functions for siRNA factors	Reference
	Dcr-2	
Drosophila flies	Expression of Vago following RNA virus infection is dependent on the helicase domain of Dcr-2	Deddouche et al. (137)
Drosophila flies	Decrease in resistance to starvation, oxidative stress and low temperature in Dcr-2 mutants Reduced lifespan in Dcr-2 mutants	Lim et al. (138)
Drosophila flies	Protein interactome reveals Dcr-2 as regulator of protein translation Protein interactome during RNA virus infection identifies antiviral proteins	Nadimpalli et al. (139) Rousseau et al. (136)
Drosophila embryos	Regulation of translation of *Toll* mRNA	Coll et al. (140)
Drosophila flies and S2 cells	Binding to chromatin and role in global transcriptional repression after heat-shock	Cernilogar et al. (135)
Aedes mosquitoes	Induction of immune-related genes, impairment of reproduction and increase of lifespan after induction of Dcr-2 or R2D2 over-expression in midgut	Dong et al. (141)
Aedes Aag2 mosquito cells	Interaction of antiviral protein aBravo with Dcr-2	Varjak et al. (142)
Culex Hsu mosquito cells	Expression of Vago following RNA virus infection is dependent on Dcr-2	Paradkar et al. (143) Paradkar et al. (144)
	Ago-2	
Drosophila flies and S2 cells	Binding to chromatin and role in global transcriptional repression after heat-shock	Cernilogar et al. (135)
Aedes mosquitoes	Reduced histone expression and inhibition of autophagy in *Ago-2* mutants	Dong and Dimopoulos (145)
Aedes Aag2 mosquito cells	Interaction of antiviral protein aBravo with Ago-2	Varjak et al. (142)

More detailed information can be found in the main text.

## 5 Aberrations in the (antiviral) siRNA pathway: uncoupling of Dicer-2 and Ago-2 function, involvement of piRNAs, interference from the covert virome, pleiotropic effects

In this section, it will be illustrated that many deviations were reported with respect to the canonical coupling of Dcr-2 (dicing) to Ago-2 (slicing) as a gene silencing/viral defense mechanism (summarized in **Table 3**). These deviations can also be expected to occur in the processing of insecticidal dsRNA for silencing of essential target genes during pest control. In addition, Dcr-2 and Ago-2 can have functions that are not directly related to the siRNA pathway of antiviral defense (summarized in **Table 2** and further discussed in section 6).

**Table 3:** Overview of aberrations in the (antiviral) siRNA pathway.

Species	Aberration or limited role of siRNA pathway in antiviral defense	Reference
Drosophila flies	No antiviral activity of siRNA pathway against persistent Nora virus infections	Habayeb et al. (146)
Drosophila flies	Antiviral activity of Dcr-2 higher than Ago-2	Han et al. (147)
Drosophila flies and S2 cells	Production of VSRs during RNA virus infections (FHV, DCV, CrPV and others)	Bronkhorst and Van Rij (33)
Drosophila S2 cells	VsiRNAs not loaded in RISC complexes during persistent virus infections	Flynt et al. (148)
Aedes mosquitoes	Antiviral activity against Zika virus by Dcr-2 and not by Ago-2	Varjak et al. (149) Harsh et al. (150)
Aedes mosquitoes	Antiviral activity of piRNA pathway, in addition to the siRNA pathway, against arbovirus infection	Varjak et al. (151)
Aedes mosquitoes	Dcr-2 deficiency has limited impact on antiviral defense	Maringer (152)
Aedes mosquitoes	Defective siRNA response against oral infection by flavivirus, for which the dsRBP Loqs2 is required	Olmo et al. (95)
Aedes U4.4 and Aag2 cells	Production of abundant vsiRNAs from defective viral RNAs that saturate the RNAi machinery	Siu et al. (153)
Bombyx Bm5 cells	Antiviral activity of piRNA pathway (dependent or independent of piRNAs), in addition to siRNA pathway, against persistent RNA virus infections	Katsuma et al. (68) Santos et al. (154) Katsuma et al. (155)
Shrimp	Conversion of dsRNA into siRNA by Dcr-2 consumes ATP and activates transcriptional response by FOXO	Gao et al. (156)

More detailed information can be found in the main text.

In *Drosophila*, where the role of RNAi genes can be assessed through well characterized mutants, following infection of a virus that does not encode a VSR, the mortality of *Dcr-2* mutants was higher than for *Ago-2* mutants (147), which points to antiviral effects of Dicer-2 in the absence of Ago-2 and, presumably, RISC formation.

During persistent viral infections in S2 (*Drosophila*) cell lines, the vsiRNAs that were produced were not loaded in RISC complexes and did not have silencing activity against reporter constructs (148). In addition, latent viral infections may be controlled in the absence of RNAi (146). Nevertheless, in most cases it is observed that Ago-2 contributes to the defense against latent infections. In mosquitoes, while both Dcr-2 and Ago-2 act anti-virally against many arbovirus infections (157), infections of Zika virus (*Flaviviridae*) are mostly targeted by Dcr-2 and not by Ago-2 (149, 150). In addition, the piRNA pathway, mainly through the action of Piwi-4, contributes to the antiviral defense in mosquitoes (151, 158).

Interestingly, in lepidopteran cell lines, both siRNA and piRNA pathways contribute to antiviral defense against the RNA virus Macula-like latent virus (MLV; related to *Tymoviridae*), although the effects of over-expression and silencing of key factors (Dcr-2, Ago-2, Siwi, BmAgo-3) on viral replication are modest (68, 69, 154, 155, 159). However, the production of vpiRNAs during MLV infection was not always observed, in contrast to vsiRNAs, which were observed during all latent infections of lepidopteran cell lines (69). It is proposed that the role of Ago-2 and RISC may be more pronounced during the early stages of pathogenic infections when a large increase in replicating viral genomes occurs while latent infections could be controlled by direct dicing of dsRNAs and other RNA degradation/immune pathways (148). However, during particular RNA virus infections of insects, the increase in vsiRNAs occurs concomitantly with the increase in viral genomes (50, 160), possibly indicating a defect in the RNAi response at the level of RISC function regarding the clearing of the viral infection. Production of siRNAs at high levels by Dicer-2 may change the cellular physiology. The processive function of Dicer-2 (see section 7) consumes ATP (161, 162) and may activate AMP-activated protein kinase (AMPK) and the transcription factor Forkhead box O (FOXO) (156). The transcriptional response of FOXO includes *Ago-2* and *Dcr-2* as well as other innate immune genes (163, 164). While such mechanism has mainly been elucidated in *Drosophila*, mosquitoes and shrimp, the findings nevertheless point to functional roles of Dicer-2 that are not strictly related to the RNAi mechanism and remain to be explored in other insects. Furthermore, the conversion into siRNAs will reduce the amount of dsRNA available for the interaction with other immune pathways, as demonstrated in mammals (165) (see also further discussion of dsRNA as PAMP in section 7).

During latent infections, defective interfering RNAs (subviral replicons) may become the source of large amounts of vsiRNAs that could act as decoys that saturate the RNAi machinery (153). However, such interference mechanism seems less likely when the much larger amounts of endo-siRNAs in cells are taken into account (148). On the other hand, there is increasing evidence that viral dsRNA enters the siRNA pathway by a different mechanism as endogenous or injected *in vitro* synthesized dsRNA, suggesting that vsiRNAs could act separately from endo-siRNAs (see section 7). From this point of view, it could be hypothesized that persistent virus infections may have a more significant impact on the exo-RNAi pathway, e.g. by the production of VSRs or induction of an “antiviral state” (166, 167; see also below for discussion of cGAS/STING pathway in section 7). Recent high-throughput sequencing efforts revealed the abundance of the virome within insects of which the majority consist of RNA viruses that have no clearly visible effects on their hosts (referred to as “covert” viruses; 42, 168–170). The impact of the covert virome on the insect innate immune response, including RNAi, remains an important topic for future research (e.g. 171, 172). Such effects nevertheless may have great variability and depend on specific virus-host infection conditions. A long-term equilibrium between persistent viruses and insect host cells may become established: in lepidopteran cell lines, silencing efficiency in RNAi reporter assays is not affected by persistent infection of Flock house virus (FHV; *Nodaviridae*) and MLV (173).

In *Ae. aegypti* mosquitoes, CRISPR-Cas9-mediated deficiency in *Dcr-2* had relatively limited impact on arbovirus infections (152). While Dcr-2 could limit viral loads in the midgut and the dissemination to other tissues, no effect on the transmission potential to vertebrate hosts was observed (37, 174). Transgenic mosquitoes that show induced expression of Dcr-2 or R2D2 in the midgut following a blood meal, nevertheless showed increased protection against Dengue virus 2 (*Flaviviridae*) or Chikungunya virus (*Togaviridae*) infection, which was attributed to the augmentation of the activity of the siRNA pathway (141). However, additional effects were observed, including the induction of many immune-related genes, including the antiviral gene Vago, as well as the impairment of reproduction and the increase of the lifespan (141). Although fewer 21 nt siRNAs were produced in *Dcr-2* mutant *Ae. aegypti*, the loss of siRNAs could not be correlated with an increase in transposon mobilization or compensatory activation of the piRNA pathway (175). Also in *Drosophila*, the role of *Ago-2* in transposon control is considered more important than *Dcr-2* (176). The non-RNAi function of Dcr-2 in the regulation of translation is elaborated in the next section.

In *A. aegypti Ago-2* mutants, arbovirus infection levels were significantly increased resulting in extensive tissue damage and high mortality (145). However, besides the disruption of the siRNA pathway, histone gene expression was reduced leading to the impairment of the DNA repair mechanisms and the repression of autophagy (145). The defective removal of damaged organelles and abnormal nuclear function contribute to the increased virulence and mortality observed during arbovirus (flavivirus and alphavirus) infections in *Ago-2* mutant mosquitoes. Thus, while *Ago-2* may have a more important role in the antiviral defense than *Dcr-2* in *Ae. aegypti*, plenty of pleiotropic effects are observed in both types of mutants.

## 6 RNAi-independent function of Dicer-2: regulation of translation

Besides an essential role in RNAi for the control of parasitic nucleic acids, Dicer-2 has also emerged as a key regulator of other cellular processes such as stress resistance (138). To have a more general overview of the multiple functions of Dcr-2 in flies and mosquitoes, immunoprecipitation experiments were carried out and its protein interactome was determined by mass spectrometry (136, 139, 142). In addition, RNAi screens were carried out to validate the function of the identified interacting proteins. Comparison of the Dcr-2 interactome in the absence or presence of viral infections has led to the identification of new proviral and antiviral host factors (136). Another approach consists of the use as baits of nucleic acids that are known as PAMPs (e.g. dsRNA) for the detection of interacting proteins during the course of viral infections (177).

The protein interactome in *Drosophila* has confirmed Dcr-2 as a regulator of protein translation, i.a. through the regulation of cytoplasmic poly-adenylation. Many interacting proteins in (female) fly extracts indicate a role for Dcr-2 in the mRNA localization mechanism during oogenesis, conform with the observed decreased fertility of Dcr-2 mutants (136). In S2 cells, interactions of Dcr-2 were confirmed with eIF4G1, a protein translation initiation factor; Rin, belonging to the GTPase activating protein (SH3 domain) binding protein (G3BP) family and component of stress granules; and Me31B, an ATP-dependent RNA helicase involved in translational repression (also known as DDX6). Previously, a role for Dcr-2 (but not Ago-2 or R2D2) was demonstrated in the regulation of translation of *Toll* mRNA through the binding of its 3’-UTR and the recruitment of a non-canonical cytoplasmic poly-adenylation mechanism involving Wispy polymerase (139, 140). In this process, Dcr-2 emerges as a regulator of Toll signaling and the innate immune response against bacteria (178).

During Dcr-2 interactome analysis, changes in interactions were observed in helicase and RNase III mutants and infections with RNA virus increased the number of protein interactions (136). Following functional analysis, new antiviral host factors were identified in *Drosophila* such as Rin and Rumpelstiltskin (Rump), a member of the heterogeneous nuclear ribonucleoprotein (hnRNP) M family (136) while Rm62, a DEAD-box helicase of the DDX5/Dbp2 subfamily and another Dcr-2 interacting protein, previously was also found to have antiviral effects (179). A similar approach in *A. aegypti* mosquito cells resulted in the identification of the new antiviral protein aBravo (**a**edine **br**oadly active **a**nti**v**iral pr**o**tein) (142). However, while aBravo interacted with Dcr-2, Ago-2 and Piwi-4 in mosquito cells, its antiviral activity was also demonstrated in Dcr-2-defective cells, indicating Dcr-2 independent function (142).

*Drosophila* Dcr-2 mutants have decreased resistance to several stresses such as starvation, oxidative stress and low temperature as well as reduced lifespan (138). The reduced triglyceride content and circulating carbohydrate levels were suggested to be caused by a dysregulation of the endo-siRNA pathway that could result in changes in expression of multiple target genes involved in the regulation of energy homeostasis (138). In contrast to Dcr-1 and Ago-1, Dcr-2 and Ago-2 are associated with chromatin and may be required for the correct execution of the global transcriptional repression after heat shock and the re-localization of elongating RNA polymerase II to heat-shock genes (135). Such process may occur by an RNAi mechanism after the production of small RNAs at the promoters of the heat-shock genes.

When the pleiotropic functions of Dcr-2 are considered, it can be asked how the activity of this multifunctional “hub” protein is regulated and to what extent its function in protein translation may interact (from interference over independence to synergism) with its function of dsRNA binding and processing. In the following section, the interaction of Dcr-2 with dsRNA and the regulation of the dicing mechanism is examined in more detail (together with the interaction of dsRNA with other immune pathways).

## 7 DsRNA as a pathogen-associated molecular pattern: activation of RNAi as well as other innate immune signaling pathways

Conform to a function as a PAMP, injection of dsRNA in the body cavity induces the expression of *Dcr-2* and *Ago2* in tissues of the silkworm and other lepidopteran insects (180–182). The induction of *Dcr-2* and other siRNA genes by dsRNA is considered an evolutionary ancient response in insects (183, 184). Besides the interaction with Dcr-2, it was recently observed that dsRNA can interact with other innate immune signaling pathways, more specifically cGAS/STING and Toll, as discussed below. Other pathways cannot be excluded, e.g. Thousand and one (TAO) kinase in *Drosophila*, a Ste20p-related serine/threonine protein kinase, was shown to interact with dsRNA and to have antiviral activity against Drosophila C virus (DCV; *Dicistroviridae*) (177). TAO kinases have pleiotropic functions and are important regulators of cellular stress, division and motility with roles in immunity and neurodevelopment (185).

### 7.1 DsRNA as PAMP: “self” versus “non-self” features

Insects generally have two Dicer enzymes of which Dcr-1 is specialized in the processing of miRNAs and Dcr-2 has evolved for antiviral defense (8). A major difference between Dcr-1 and Dcr-2 is the helicase domain at the N-terminus which is conserved and hydrolyzes ATP in Dicer-2 but has become degenerate in Dicer-1 (186).

Since large amounts of endogenous dsRNA are produced in cells and dsRNA can act as a PAMP, the question arises how the activation of the innate immune response can be prevented. In mammals, such mechanisms were investigated in more detail and include modification by Adenosine Deaminase Acting on RNA (ADAR) enzymes, pseudo-uridylation and methylation (e.g. N6-methyladenosine (m6A)) and attachment of the 5’-CAP (187, 188). Furthermore, nuclear export of genome-derived dsRNA is needed for the interaction with cytoplasmic dsRNA sensors, which can be prevented by degradation by nucleases in the nucleus such as the nuclear RNA exosome and potentially Drosha (188). The miRNA pathway is constructed as to promote its recognition as “self”: the primary miRNAs with longer hairpins are processed in the nucleus while the pre-miRNAs that are transported through the nuclear pores are mono-phosphorylated and display only short dsRNA structures with typical mismatches (188). Recent studies indicated a role for ADAR in the response against BmNPV in the silkworm (189). *BmADARa* expression is strongly induced after BmNPV infection and over-expression/silencing experiments support an antiviral role. Inhibition of BmNPV was proposed to be enhanced through the interaction of BmADARa with the DEXHc domain of BmDcr-2 (189). In *Drosophila*, loss of *ADAR* results in the induction of various innate immune genes which could be rescued by RNAi of *Dcr-2* (190). It is assumed that dsRNA editing by ADAR could result in weakened recognition by the helicase domain of Dicer-2 which functions as a cytosolic sensor for dsRNA to trigger immune signalling, independent of dicer enzymatic activity (137) (see section 7.3).

For the purpose of pest control, it may be important to investigate whether administrated dsRNA will be recognized as “self” or “non-self”. More research is needed which mechanisms contribute to the different recognition of endogenous versus exogenous dsRNA.

### 7.2 Specific mechanisms for recognition of dsRNA by Dcr-2 for production of siRNAs

#### 7.2.1 Interaction with the helicase domain

The helicase domains of Dicers belong to the subgroup of the retinoic acid-inducible gene-I-like receptors (RIG-I-like receptors or RLRs), which function as pathogen pattern recognition receptors for dsRNA in vertebrates (191). The mechanism by which (*Drosophila*) Dcr-2 cleaves dsRNA substrates is dependent on their ends: in the case of blunt ends, an optimal interaction occurs which results in ATP hydrolysis driving processive cleavage, while for 3’-overhangs a distributive cleavage occurs that does not require ATP and after which Dcr-2 dissociates (192, 193). The difference is explained by the preferential recognition of blunt dsRNA by the helicase domain while 3’-overhang ends are recruited by the PAZ-platform module. Viral dsRNA is proposed to contain blunt ends and therefore is recognized by the helicase domain of Dcr-2 as “non-self” for initiation of multi-cleavage processing; dsRNA structures with 3’-overhangs on the other hand resemble “self” miRNA precursors for single cleavage (162, 186). While Dcr-2 is fully functional for the recognition of viral dsRNA, the specialization of Dcr-1 to miRNA processing is reflected in its degenerate helicase domain (186). The importance of the helicase domain of Dcr-2 for silencing, 21 nt siRNA production and antiviral defense against Semliki forest virus (SFV; *Togaviridae, Alphavirus*) was also demonstrated in a Dcr-2 knock-out cell line from the mosquito *Ae. aegypti* (194). Mutations located in motifs involved in ATP binding and hydrolysis revealed the crucial role of the helicase domain of Dcr-2 in antiviral RNAi, presumably through the promotion of processive viral dsRNA cleavage (194). As a mechanism, it was envisaged that the cleavage initiated at the blunt terminus of a long dsRNA molecule generates the first dsRNA terminus with a 2 nt overhang that will be used by the PAZ domain to allow the production of 21 nt vsiRNAs of precise size (195). However, it remains to be determined to which extent dsRNA molecules with blunt ends are produced during viral infection *in vivo*. The question indeed arises how the termini of the viral dsRNAs become recognized for efficient processing, especially when it is often observed that such termini are protected. For instance, viral protein genome-linked (VPg) proteins that are covalently attached to the 5’-end of positive-strand ssRNA viruses play an essential role in their protein-primed replication (196). Another factor relates to the existence of persistent viruses in insects (e.g. representing an unknown virome in each insect), that could modulate the function of Dcr-2. As already discussed, several VSRs in insect viruses are dsRNA-binding proteins (dsRBPs) that prevent the interaction of viral dsRNA with Dicer (33, 197). Notably, detection of VSRs themselves as PAMPs may evolve as a counter-counter-defense mechanism to trigger a non-canonical immune response (198), as also observed in plants (199).

For the application of insecticidal dsRNA, the structural ends of dsRNA may need to be considered to guarantee the maximal stimulation of Dicer activity. More research needs to be carried out on Dcr-2 enzymes of different insects regarding the rules of recognition of dsRNA for optimal processing into siRNAs.

#### 7.2.2 The role of small dsRNA-binding proteins

In addition, the two Dicers interact with different accessory dsRNA-binding proteins (dsRBPs) through their helicase domains (186). In *Drosophila*, the Loquacious (Loqs) PA and PB isoforms interact with Dcr-1 and affect miRNA processing (200). The Loqs-PD isoform, on the other hand, is required for the formation of endo-siRNAs and the processing of injected *in vitro* synthesized dsRNA into siRNAs (201, 202). Strikingly, however, Loqs-PD is not required for processing of viral dsRNA (produced during viral infection) into vsiRNAs (202) and *in vitro* studies have indicated that Loqs-PD enables the cleavage of suboptimal substrates such as dsRNAs with blocked, structured or frayed ends (203, 204) that are present in cellular endogenous siRNA precursors or dsRNA typically synthesized *in vitro* by T7 RNA polymerase from PCR-generated DNA substrates (35). Thus, Loqs-PD is an important modulator to allow the cleavage irrespective of the type of dsRNA termini while it is not required for the antiviral response.

Another dsRBP, R2D2, functions downstream of processing of long dsRNAs and is required for the loading of siRNAs (both endo-siRNAs and vsiRNAs) into Ago-2 in flies (202). In *Drosophila*, R2D2 co-localizes with Dicer-2 to cytosolic foci (D2 bodies; 205) and prevents the cleavage of non-canonical pre-miRNA substrates by Dicer-2 *in vitro* (14). However, during screening in S2 cells, R2D2 and even Dcr-2 were dispensable for silencing triggered by exogenous dsRNA, in contrast to Ago-2 (206). In lepidopteran cell lines, expression of R2D2 is low or absent while efficient RNAi is observed after transfection of dsRNA (207). In locusts, both Dcr-1 and Dcr-2 enzymes contribute to efficient gene silencing triggered by exogenous dsRNA, indicating at least partial independence of gene silencing from the antiviral function of Dcr-2 (208). Such observations indicate considerable flexibility regarding the pathways that recruit (non-viral) exogenous dsRNA for processing to trigger RNAi. Also in mosquitoes long hairpin RNAs can be processed by both Dcr-1 and Dcr-2 for the production of 21 nt siRNA or 22–23 nt miRNA, respectively (209) (see further discussion below).

The distinctiveness of the antiviral dsRNA trigger was emphasized when the mechanism of viral dsRNA production was elucidated during DNA virus infection. During IIV-6 infection in *Drosophila*, viral dsRNAs become synthesized in hot-spot AT-rich regions of the viral genome by the host RNA polymerase II (RNAPII) complex (210). However, conditions for viral dsRNA production are different than for endo-siRNA precursors since a non-canonical RNAPII complex is involved that lacks positive transcription elongation factor b (P-TEFb) (210). In addition, Loqs-PD is not required for production of vsiRNAs during IIV-6 infection. Transcription elongation in the presence of P-TEFb is correlated with the presence of the m^6^A methylation modification in the endo-siRNA precursors that can be interpreted as a “self” motif to avoid cellular toxicity (210). This finding reinforces the view about the special conditions during which the antiviral siRNA pathway is activated.

The Loqs-PD isoform however seems not to be conserved in other insects outside of *Drosophila*. In the mosquito *A. aegypti*, R2D2 is involved in the formation of exo- and endo-siRNAs while Loqs-PA is specific for processing of miRNAs; the Loqs-PB isoform plays a more complex role by regulating the three types of small RNAs (211, 212). However, a paralog of both R2D2 and Loqs, Loqs2, was identified in *Aedes* (but not *Culex* or *Anopheles*) mosquitoes that was shown to mediate the systemic antiviral RNAi response against arbovirus (flavivirus) infections (95). The absence of expression of Loqs2 in the midgut was correlated with a defective siRNA response against oral infection by Dengue virus (see also section 3.3). Interestingly, *Ago-2* and other canonical siRNA genes are expressed in the midgut of *A. aegypti* that is fully functional when activated by endogenous and exogenous dsRNA sources, indicating that Loqs2 specifically affects antiviral silencing (95). Loqs2 protein preferentially localizes to the cellular nucleus, shows specific expression in reproductive tissues and embryos and its ectopic expression causes developmental arrest in larvae, indicating neo-functionalization driven by positive selection (213). On the other hand, independent of its activity as RNAi factor, Loqs-PA was identified as a proviral host factor for replication of Dengue virus and other flaviviruses in Aag2 cells (212, 214). Loqs-PA interacts with NS1 and other non-structural viral proteins and becomes recruited to the 3’-UTR of Dengue virus for the facilitation of viral RNA replication (212, 214).

In *Diabrotica*, high expression of Loqs was observed compared with other species such as *Spodoptera* (Lepidoptera) and southern green stink bug (Hemiptera), which was speculated to be a contributing factor for the high sensitivity to environmental RNAi (215). While also in coleopteran transcriptomes no Loqs-PD isoform was found, another dsRBP, StaufenC, was identified in *L. decemlineata*, whose function was critical for RNAi triggered by exogenous dsRNA (216, 217). At least in some coleopterans, such as *Leptinotarsa* and *Diabrotica*, that are very sensitive to environmental RNAi, StaufenC localizes to the endoplasmic reticulum (ER) of the cells where it regulates the uptake of dsRNA into the cytosol through interactions with proteins from the ER-associated degradation (ERAD) pathway (118). In the BCIRL-Lepd-SL1 coleopteran cell line, dsRNA is taken up by endocytosis but then becomes trafficked by the recycling pathway to the Golgi network and subsequently to the ER by retrograde transport (118). The role of StaufenC in the production of exo-siRNAs was found to be much more important than Loqs or R2D2. A possible function for StaufenC in antiviral defense has not been explored yet.

In *Locusta migratoria*, three isoforms of Loqs (Loqs-PA, Loqs-PB and Loqs-PC) were identified, which are involved in the miRNA and both endo- and exo-siRNA pathways (218). Over-expression of the locust Loqs proteins in *Drosophila* S2 cells resulted in isoform-specific effects: LmLoqs-PB could affect positively all three pathways while LmLoqs-PA and LmLoqs-PC could stimulate specifically silencing by exo- and endo-siRNAs, respectively. A role for R2D2 in the *Locusta* siRNA pathway was also demonstrated (219). The role of specific dsRBPs in antiviral defense in locusts has not been reported hitherto.

In Lepidoptera, one Loqs gene/protein was described that has functions in both miRNA and (exogenous and endogenous) siRNA pathways (207, 220). In the fall armyworm, sequences corresponding to both Loqs-PA and -PB isoforms were found (215). An isoform that has replaced the third dsRNA-binding motif with unique sequence, similar to Loqs-PD, apparently was also present in *S. frugiperda*. As mentioned before, it was observed that R2D2 was expressed at low levels in the silkworm, *B. mori*, and lepidopteran insects in general (221, 222). A comparative analysis of RNAi factors indicated that the Lepidoptera present the highest phylogenetic distance from the other insect orders (167). Perhaps paradoxically, the greatest variability was observed in elements of core factors in the miRNA pathway which could somehow influence RNAi sensitivity in the Lepidoptera.

In the above discussion, the crucial role of dsRBPs in the siRNA pathway is revealed. Blunt dsRNAs may not need specific dsRBPs for efficient processing while for endocytosed dsRNAs membrane-localized dsRBPs like StaufenC seem to be required for efficient translocation to the siRNA machinery in the cytoplasm. In mosquitoes, a specialized dsRBP is required for antiviral defense, illustrating the idiosyncrasies in the evolution of RNAi. Interestingly, in some species exogenous dsRNA may be processed by Dcr-1 and Dcr-2, again demonstrating mechanism variability among insects. For the evaluation of the activity of dsRNA in particular species, useful information could be obtained by the determination of the protein interactome against dsRNA (e.g. 223). Identification of dsRBPs could give clues about the pathway of uptake and processing of dsRNAs.

#### 7.2.3 Other factors that may affect the efficiency of dsRNA processing and siRNA function

For an alphanodavirus that persistently infects Hi5 cells, a lepidopteran cell line derived from *Trichoplusia ni*, a phased pattern of vsiRNAs with periodicity of 20 nt was observed, indicating that Dcr-2 in Lepidoptera is a processive enzyme like in *Drosophila*, capable of translocating along the dsRNA substrate in an ATP-dependent manner (224). The processive pattern of cleavage was also detected during BmCPV infection of the midgut in *B. mori* (50). An interesting observation is also that siRNAs in Lepidoptera are not 2’-O-methylated, in contrast to siRNAs in *Drosophila* and to piRNAs (225), with no obvious effects on 3’-end stability or Ago-2 loading (224). Nevertheless, dsRNA substrates are vulnerable for degradation by nuclease enzymes, e.g. by RNAi efficiency-related nuclease (REase) in Lepidoptera, which could interfere with the function of Dicer (226). In the Asian corn borer (*Ostrinia furnacalis*) and the cotton bollworm (*H. armigera*), dsRNAs become cleaved at GGU-sites, which was not observed in the coleopteran *Tribolium* and may play a role in gene silencing efficiency (227). Cleavage of dsRNA that was not directly related to the RNAi machinery occurred during dsRNA virus infection of the silkworm midgut as well (50).

In conclusion, when dsRNA is internalized into the cytoplasm, competition between dicing and other degradation pathways may be expected, which can be revealed by patterns in analysis of data from small RNA sequencing analysis. On the other hand, efficient processing of dsRNA into siRNAs also does not guarantee an efficient silencing response, as already discussed in section 5. The combination of assessment of small RNA production in combination with RNA degradome sequencing (228) should give a more holistic view of the processing of dsRNA produced during viral infection, and during the application of insecticidal dsRNA as well.

### 7.3 Dcr-2 as a viral sensor for the activation of immune pathways

The role of RNAi during virus infection in *Drosophila* and the silkworm has already been discussed above (sections 2 and 3). However, the occurrence of alternate pathways by which viral dsRNA interacts with Dcr-2 as opposed to other exogenous and endogenous dsRNA substrates remains to be clarified.

In *Drosophila*, Vago, a small, secreted protein with a conserved single “von Willebrand factor type C” domain and antiviral function, was induced during DCV and Sindbis virus (SINV; *Togaviridae, Alphavirus*) infection, which required an intact DExD/H-box helicase domain of Dcr-2 (11, 137). Expression of the secreted peptide CxVago in a Dcr-2-dependent manner was also detected after infection of West Nile virus (*Flaviviridae*) in the Hsu cell line, derived from the mosquito *Culex quinquefasciatus* (143, 144, 158). CxVago was found to activate the Jak-STAT pathway to induce the target gene *vir-1* and to inhibit West Nile virus (*Flaviviridae*) replication in Hsu cells. In shrimp, Vago-like peptides may play multiple roles in antiviral defense and can adopt functions as both signaling molecules and pathogen sensors (229). In *Drosophila*, the Jak-STAT may not be considered as a “true” antiviral defense pathway (conform to the interferon system in vertebrates) since its response may be primarily caused by the detection of cell damage *per se* (230, 231). While the connection of the Jak-STAT pathway to antiviral defense may be indirect because of its general involvement in tolerance to tissue damage by the activation of repair mechanisms (232), recent research in shrimp and hemipteran insects confirms a more complicated picture in which the Jak-STAT pathway may function directly in the detection of pathogens (including viruses) for the activation of anti-bacterial or antiviral effector genes (233, 234).

In summary, the function of the helicase domain of Dcr-2 as a sensor that detects virus-produced dsRNA for the activation of a transcriptional response of immune genes deserves further investigation about its occurrence in other insects. The potential interaction between the function of Dcr-2 as sensor for signaling and its role as dicing enzyme also remains to be clarified on an insect-specific basis.

### 7.4 Activation of antiviral defense by the cGAS-STING pathway: interaction/interference with RNAi?

Until recently, the existence of interferon (IFN)-like signaling pathways that control the expression of IFN-stimulated genes (ISGs) for the control of viral infections was considered controversial in insects. However, with the discovery of the ancient function of the cyclic GMP-AMP synthase (cGAS)/Stimulator of interferon (STING) pathway, it has become apparent that invertebrates encode the capacity to respond to virus detection with transcriptional responses that include antiviral effector genes (133, 134, 235, 236). Also the Jak-STAT pathway is increasingly considered to have such a function as discussed above (section 7.3).

In *Drosophila*, three cGAS-like receptors (cGLRs) were identified of which both cGLR-1 and cGLR-2 respond to dsRNA while the ligand for cGLR-3 remains unidentified (237–239). Diverse cyclic di-nucleotides (CDNs, such as 2’3’-cGAMP, 3’2’-cGAMP, 2’3’-c-di-AMP, 2’3’-c-di-GMP) produced by cGLRs then interact with STING (240) which results in the activation of the NF-κB transcription factor Relish through the kinase IKKβ which is part of the IMD innate immune pathway (241). STING-regulated genes (Srgs) include STING itself (comprising a positive feedback loop) and Nazo, an antiviral effector with specificity against picorna-like viruses such as DCV and Cricket paralysis virus (CrPV) (241).

Importantly, local activation of the cGLRs and STING leads to the production of a systemic signal, likely corresponding to CDNs, that confer antiviral defense in secondary tissues, mainly the central nervous system, the midgut, the Malpighian tubules and the reproductive ducts (239). In a comparative analysis, injection of 2’3’-cGAMP in adult flies resulted in a transcriptional response in which the number and identity of putative Srgs was variable among 10 different *Drosophila* species although it included previously identified antiviral genes such as *pastrel*, the siRNA factors *Ago-2* and *Dcr-2* and the secreted protein Vago (242). Srgs have tissue-specific expression since Srg-1 (with unknown function) and Srg-2 (encoding a glucose transporter) are produced by the fat body and midgut epithelial cells, respectively, while Srg-3 (unknown function) has more ubiquitous expression (243). Among Srg-1-3, only Srg-3 has antiviral effects against DCV and Rift valley fever virus (RVFV; *Phenuiviridae*) but not Vesicular stomatitis virus (*Rhabdoviridae*) or Dengue virus (*Flaviviridae*) (243).

The available evidence therefore indicates that cGLRs correspond to PAMP-recognition receptors (PRRs) that detect dsRNA and generate CDNs for the activating of STING signaling and the production of antiviral effectors after the induction of a transcriptional response. In addition, CDNs can be exported outside the cells and may act systemically to induce an antiviral state throughout the body. It is noted that also many bacteria naturally produce CDNs and activate the STING pathway as part of the innate immune response (244). Furthermore, the STING pathway can also control virus infection through the regulation of autophagy (245). In general, STING signaling may be involved in a plethora of different biological processes such as stem cell proliferation, lipid metabolism and central nervous system functions like sleep pattern (239, 246, 247).

The discovery of the antiviral function of cGAS/STING and the activation of cGLRs by dsRNA raises the question of the interaction with the exo-siRNA pathway which until recently was considered the most important antiviral defense mechanism in insects (231). The induction of Srgs by CDNs occurs in *Ago-2* mutant flies, indicating independence from RNAi (241, 243). Furthermore, *Drosophila* cGLR-1 and cGLR-2 recognize dsRNAs longer than 30 bp, which exceeds the length of siRNAs (238). On the other hand, both *Ago-2* and *Dcr-2* are induced by STING signaling although the effects are species-dependent (242). Additional research is required with respect to the extent of cooperation/competition between the RNAi and cGAS/STING pathways and whether their relative importance may depend on parameters such as tissue and developmental specificity or type of virus infection.

STING homologs appear to be present in all major insect orders including Diptera, Lepidoptera, Coleoptera, Hemiptera, Hymenoptera, Orthoptera and Blattoidea but may be absent in lineages such as mosquitoes (231, 235). It is striking that Lepidoptera are characterized by an expansion of the poxin genes that encode nucleases that cleave CDNs (236). Poxin genes are also present in several viruses that infect lepidopteran insects such as baculoviruses, granuloviruses, cypoviruses and entomopoxviruses. In silkworm (*B. mori*) cells, an antiviral function of BmSTING was demonstrated against BmNPV infection through a mechanism that involved the activation/cleavage of BmRelish and the production of antimicrobial peptides (AMPs) such as cecropin and gloverin (248). In addition, a negative regulator of the STING pathway, BmCasp8L, was identified that interacts with STING and prevents the activation of Relish by Dredd caspase (248). With respect to BmNPV, silencing of its poxin gene by RNAi resulted in effects on budded virus production and viral gene expression (249). Besides poxin proteins that accumulate intracellularly and degrade 2’3’-cGAMP, baculoviruses may also encode poxins that are secreted (250). Interestingly, besides 2’3’-cGAMP degradation activity, extracellular poxins can also act as inhibitors of melanization, presumably through the inhibition of prophenoloxidase activation (250).

### 7.5 Insect Toll receptors as PRRs

During phylogenetic analysis, Toll receptors that are compared between mammals and insects typically separate into two groups: (1) mammalian Toll-like receptors (TLRs) which cluster together as PRRs that are activated by PAMPs from micro-organisms; and (2) insect Toll receptors which are generally considered as cytokine receptors, as exemplified by *Drosophila* Toll-1 which is activated by extracellularly processed Spätzle, a secreted protein with cystine-knot domain (251). Insect Toll-9 receptors, however, form an exception since they resemble more closely mammalian TLRs with respect to both the ligand-binding ecto-domain and the intracellular Toll/Interleukin-1 receptor (TIR) signaling domain (252, 253).

Recently, DmToll-9 was reported to interact with dsRNA in endosomes and to induce the expression of the siRNA pathway genes *Dcr-2* and *Ago-2*, which results in protection against DCV (*Dicistroviridae*) infection (254). In the presence of DmToll-9, reduced AKT signaling (related to growth/survival) was observed which correlated with the induction of *Dcr-2* and *Ago-2*.

Phylogenetic analysis revealed the existence of three Toll-9 clades in insects, which may have been the ancestral condition (255). While only one *Toll-9* gene is found in *Drosophila*, two genes are found in *B. mori*, of which *BmToll9–1* belongs to the same subclade as *DmToll-9* and *BmToll9–2* is more distant (255–257). Recently, BmToll9–1 was identified as a PRR that is activated by bacterial lipopolysaccharide (LPS) in the presence of two (Myeloid differentiation-like) ML proteins that could act as co-receptors (253). However, the expression of *BmToll9–1* in the midgut of larvae can also be modulated by dsRNA, in addition to LPS (181), and expression of BmToll9–1 in silkworm-derived Bm5 cells facilitated the induction of *Dcr-2* in response to dsRNA (258). Expression of *BmToll9–2* is also regulated by dsRNA (259). The interaction of Toll-9 receptors with dsRNA deserves more attention and the possible function of Toll receptors as PRRs may also need re-evaluation, as exemplified by the discovery of the Toll5/ML-11 complex as a sensor for the detection of sphingomyelin and the baculovirus envelope in *Spodoptera litura* (260). Both BmToll9–1 and BmToll9–2 were shown to positively regulate immune responses in the silkworm. Cross-validation confirmed *BmToll9–1* and *-2* as two genetically distinct genes, suggesting potential independent regulatory mechanisms (261–263).

## 8 Discussion

RNAi was heralded as a new approach for insect pest control but it has been established for some time that large differences exist among insects with respect to sensitivity to environmental RNAi (i.e. RNAi by feeding; section 4). In addition, the use of dsRNA as insecticide is complicated by its role as trigger in antiviral defense (sections 7.3, 7.4 and 7.5). While originally the antiviral/insecticidal siRNA pathway was presented as a linear straightforward process of dicing of dsRNA and slicing of mRNA by siRNA, research over the last 25 years has uncovered many aberrations/complications in the proposed canonical pathway. This review has attempted to produce an overview of the many factors that can modulate the RNAi process that is intended to be exploited for pest control. An (hypothetical) overview of the pathways of dsRNA processing/signaling, for both exogenous RNAi and virally produced dsRNA, is provided in **Figure 1**. A more detailed (hypothetical) diagrammatic scheme of the fate of dsRNA as an immune trigger, with a comparison between RNAi-sensitive and -recalcitrant insects, is presented in **Supplementary Figure S1**.

This analysis was written to provide a detailed background about the current perceived complexity in model insects regarding RNAi and dsRNA signaling to researchers that use RNAi in silencing experiments as well as to scientists that develop RNAi-based methods for insect pest control. In general, this review could incite further research into three topics that involve RNAi in insects, as well as other arthropods and invertebrates: 1) Improvements in the design of the dsRNA trigger; 2) Elucidation of the specificity of the RNAi response; 3) Evaluation of dsRNAs as safe insecticides.

### 8.1 Improvements in the design of the dsRNA trigger

Great strides of progress have been taken in the design of dsRNA molecules to have the highest efficiency in gene silencing by RNAi (132, 264). The interaction of dsRNA with the helicase domain of Dcr-2 nevertheless has also revealed that the ends of the molecules with dsRNA structures play an important role in the activation of dicing as well as the innate immune response (section 7.2.1). In addition, dsRBPs act as catalysts not only for the resolution of the ends of the dsRNAs but also for their crossing of the plasmamembrane, a crucial barrier for uptake (section 7.2.2). Future research should therefore try to clarify how different ends of dsRNAs (e.g. blunt, 3’-overhangs, frayed, panhandles, triphosphates, virus-derived peptides, 3D folds…) could affect the route of uptake of dsRNAs and the activation of the dicing function of Dcr-2 as opposed to the non-specific induction of immunity. Dicing assays and the transcriptional response of immune-related genes could be used for evaluation. The determination of the protein interactome with dsRNA in insect pests could also increase our knowledge of its uptake/processing pathways. Furthermore, as indicated in section 7.1, dsRNA can be recognized as “self” or as “non-self”, a process which has not received much attention in insects and other arthropods/invertebrates. Thus, it could be assessed to what extent dsRNA molecules with different end structures could be targeted by ADAR enzymes (265).

### 8.2 Elucidation of the specificity of the RNAi response

As elaborated in section 4, high differences can exist with respect to both systemic RNAi (administration of dsRNA by injection) and environmental RNAi (administration of dsRNA by feeding) among insects. In many experiments that employ insects that are relatively recalcitrant to RNAi, phenotypes are often limited to the recording of (sub)lethal effects and silencing of the targeted genes, in comparison with the administration of a non-specific dsRNA, mostly targeting green fluorescent protein (dsGFP). For the development of an insecticide based on the RNAi mechanism, such information is insufficient. More information is needed regarding the processing of dsRNA to siRNAs (by sequencing of small RNAs) and the degradation of the target (by sequencing of the RNA degradome) (section 7.2.2). Additionally, the proper phenotype should be observed as would be expected from silencing of the specific target. Besides the proper activation of the RNAi mechanism, the non-specific activation of the immune/stress response by mRNA sequencing should also be checked. Indeed, the silencing of the targeted gene could be a consequence of the triggering of stress mechanisms by high concentrations of dsRNA and the use of just one particular control (dsGFP) may be inadequate to comprehensively understand the changes in physiology (e.g. immune state) that are being triggered.

With respect to the leaf beetles that are very sensitive to dsRNA by feeding and against which RNAi-based insecticides have been developed, it would be equally interesting to determine whether transcriptome responses of immune-related genes can be observed and whether such responses are dose-dependent (since low doses already cause significant gene silencing). Furthermore, little is known about the function of RNAi as an antiviral mechanism in leaf beetles and providing such missing information would contribute to the global understanding of the role of RNAi in antiviral immunity.

### 8.3 Reverberations on the evaluation of dsRNAs as safe insecticides in field trials

RNAi-based pest control continues to receive good reviews regarding safety and fits extremely well within the concept of integrated pest management (266). Our analysis nevertheless indicates that dsRNA, as an immune trigger, can have effects on insect physiology beyond specific gene silencing by the RNAi mechanism. Laboratory tests and field trials generally confirm that RNAi-based insecticides are safe to the environment, are not a threat to human health and livestock, and do not affect non-target beneficial insects (267–273). However, it may be overlooked that dsRNA may not induce RNAi in non-target species but instead, as a PAMP, may trigger long term effects in the physiology of other species that can affect the immunity against pathogens. Indeed, non-specific effects of dsRNA that can modulate the antiviral response have been reported in honeybees previously (274, 275). Progress in the understanding of the immune response triggered by dsRNA as PAMP in model insects allows now more specific predictions of the pathways that can be involved (e.g. NF-κB, cGAS-STING, Toll). Such research may require the testing of changes in the resistance/tolerance of the non-target insects against multiple pathogens as well as long term follow-up studies.

## 9 Conclusion

There is increasing evidence that the core siRNA factors may have additional functions in the stress/immune response leading to non-specific effects and that dsRNA may activate other innate immune pathways as a PAMP. Importantly, the specific features that define dsRNA as “self” or “non-self” and determine the nature of the response, hardly have been explored in insects. The integration of the exo-RNAi process in the innate immune response also indicates that the success of its application in pest control will require detailed investigations in the targeted insect pest with respect to the role of dsRNA as PAMP and the regulation of the function of Dcr-2 and Ago-2. Such considerations equally apply to the role of exo-RNAi as an antiviral defense mechanism, as indicated by the analysis in Lepidoptera. Analogous to “precision medicine” that takes into account variability among human patients, future environmentally safe approaches for pest management may require embracing the concept of “precision pest control” that takes into account population-specific differences in the regulation of stress and immunity by triggers such as dsRNA. This perspective therefore advocates an increase in efforts to understand the variability of the stress/immune response among insects for the guidance of new methods for pest control.

## Supplementary material

Supplementary Figure 1 Hypothetical diagram of dsRNA recognition/processing by innate immunity and RNAi pathways. Extracellular (insecticidal) dsRNA may have features that are recognized as either self or non-self while intracellular viral dsRNA is expected to be perceived as non-self. In permissive insects, low doses of dsRNA are taken up into the cytoplasm and processed by the canonical exo-RNAi pathway for specific target silencing and phenotype induction. DsRBPs and VSRs could modulate and interfere with this process. In addition, dsRNA may interact with PRRs such as Dcr-2 helicase domain and cGAS-like receptors to trigger NF-κB-mediated immunity. In recalcitrant insects, high doses of dsRNA also accumulate in endosomes and may stimulate Toll receptors to induce a non-specific stress response that is expected to interfere with the canonical exo-RNAi (siRNA) pathway. During virus infections, other PAMP and damage-associated pattern (DAMP) molecules can contribute to the (non-RNAi) stress/immune response.
