# Supplementary material for: DsRNA as pathogen-associated molecular pattern in innate immunity and multiple functions of the RNAi machinery complicate the use of RNAi in pest control
Source: Front Insect Sci. 2026 Jan 16;5:1749008. doi: 10.3389/finsc.2025.1749008 (PMC12855433; doi:10.3389/finsc.2025.1749008)
Supplement: Supplementary Figure 1 — Hypothetical diagram of dsRNA recognition/processing by innate immunity and RNAi pathways. Extracellular (insecticidal) dsRNA may have features that are recognized as either self or non-self while intracellular viral dsRNA is expected to be perceived as non-self. In permissive insects, low doses of dsRNA are taken up into the cytoplasm and processed by the canonical exo-RNAi pathway for specific target silencing and phenotype induction. DsRBPs and VSRs could modulate and interfere with this process. In addition, dsRNA may interact with PRRs such as Dcr-2 helicase domain and cGAS-like receptors to trigger NF-κB-mediated immunity. In recalcitrant insects, high doses of dsRNA also accumulate in endosomes and may stimulate Toll receptors to induce a non-specific stress response that is expected to interfere with the canonical exo-RNAi (siRNA) pathway. During virus infections, other PAMP and damage-associated pattern (DAMP) molecules can contribute to the (non-RNAi) stress/immune response. [file Presentation1.pptx]

## Slide 1
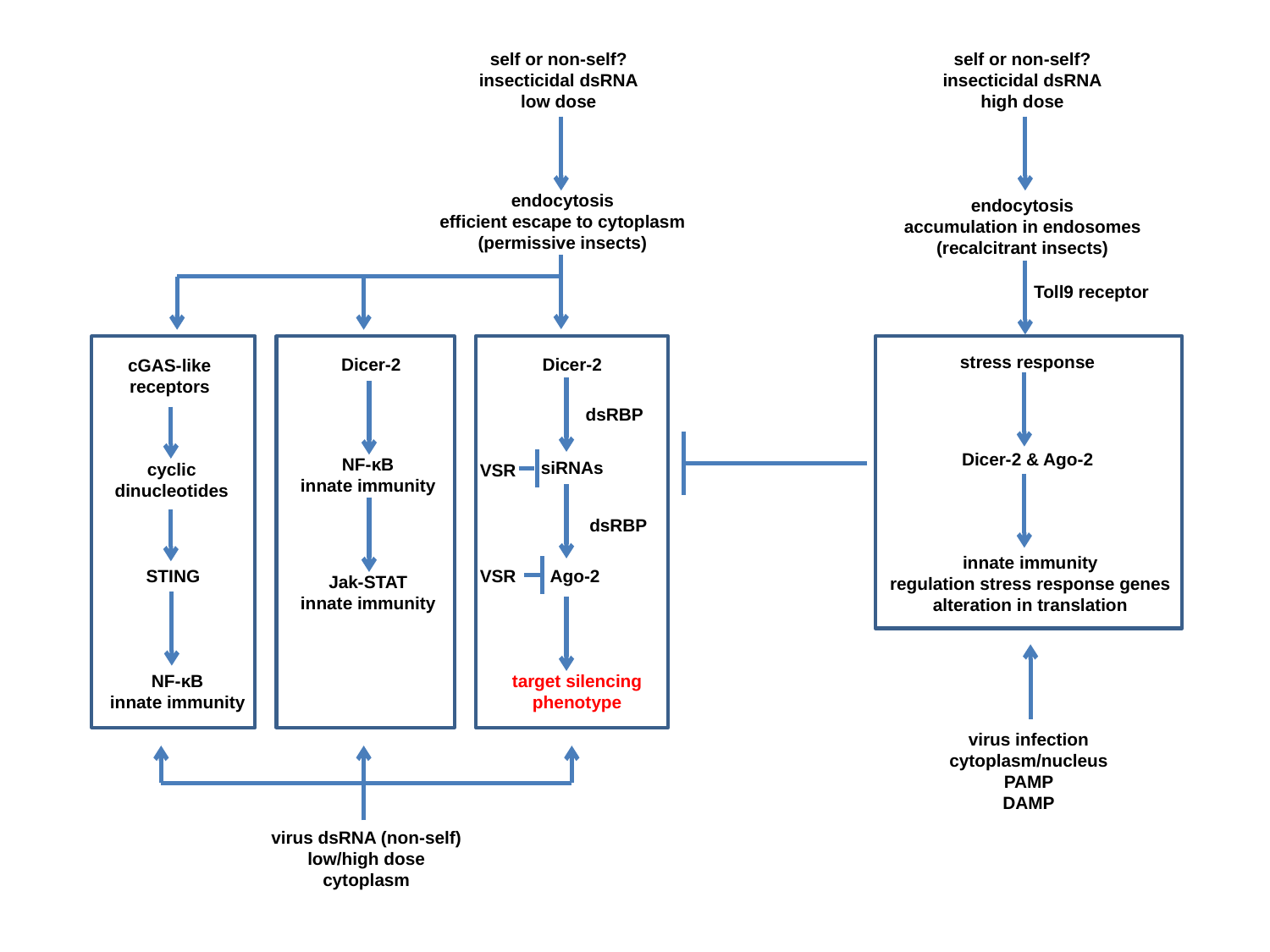

self or non-self?
insecticidal dsRNA
low dose
self or non-self?
insecticidal dsRNA
high dose
endocytosis
efficient escape to cytoplasm
(permissive insects)
endocytosis
accumulation in endosomes
(recalcitrant insects)
Toll9 receptor
stress response
Dicer-2
Dicer-2
cGAS-like
receptors
dsRBP
Dicer-2 & Ago-2
NF-κB
innate immunity
siRNAs
cyclic
dinucleotides
VSR
dsRBP
innate immunity
regulation stress response genes
alteration in translation
STING
VSR
Ago-2
Jak-STAT
innate immunity
NF-κB
innate immunity
target silencing
phenotype
virus infection
cytoplasm/nucleus
PAMP
DAMP
virus dsRNA (non-self)
low/high dose
cytoplasm
